# Supplementary material for: A review of coral bleaching specimen collection, preservation, and laboratory processing methods
Source: PeerJ. 2021 Jul 8;9:e11763. doi: 10.7717/peerj.11763 (PMC8272927; doi:10.7717/peerj.11763)
Supplement: Supplemental Information 5 — Data categories include: (1) meta-data, (2) sampling design, (3) specimen collection, (4) specimen preservation, (5) specimen processing, and (6) downstream analyses. The number of studies in each category is in parentheses. The total number of studies in each category (second column) were used as the denominator in calculating the percentages. [file peerj-09-11763-s005.docx]

| **1) META-DATA** | **Number of studies** | **Summary statistics and percentage data** |
| --- | --- | --- |
| 1. Year of publication | 171 | 15.2% 2014 (26)  17.5% 2015 (30)  16.4% 2016 (28)  14.6% 2017 (25)  15.8% 2018 (27)  8.2% 2019 (14)  12.3% 2020 (21) |
| 2. Category of study | 171 | 38.6% Bleaching surveys (66)  61.4% Bleaching experiments (105) |
| 3. Author(s) and title of publication | 171 | See Supplement 2 |
| 4. Journal of publication | 171 | Summarized in Figure S2; see Table S2 for complete journal list |
| **2) SAMPLING DESIGN** | | |
| 1. Coral family, genus, and species name | 171 | Family ^a^  55.6% Acroporidae (95)  35.7% Poritidae (61)  32.7% Pocilloporidae (56)  31.6% Merulinidae (54)  8.2% Dendrophylliidae (14)  7.0% Mussidae (12)  5.3% Agariciidae (9)  5.3% Euphyllidae (9)  4.1% Siderastreidae (7)  3.5% Faviidae (6)  2.9% Coscinaraeidae (5)  2.9% Montastraeidae (5)  2.3% Fungia (4)  2.3% Plesiastreidae (4)  1.8% Meandrinidae (3)  1.8% Oculinidae (3)  1.2% Psammocoridae (2)  0.6% Caryophylliidae (1)  See Table S3 for complete species list. |
| 2. Number of species sampled per study | 171 | 100% Reported (Mean ± 1SD = 2 ± 2; Median = 1)  62.6% one species (107)  17.5% two species (30)  7.6% three species (13)  3.5% four species (6)  1.8% five species (3)  4.1% six species (7)  2.9% between seven and eighteen species (5) |
| 3. Number of parent colonies sampled per species | 171 | 91.2% Reported (Mean ± 1SD = 17 ± 27; Median = 7) (156)  60.2% between 1 and 10 (103)  13.5% between 11 and 20 (23)  4.1% between 21 and 30 (7)  2.9% between 31 and 40 (5)  2.3% between 41 and 50 (4)  1.8% between 51 and 60 (3)  1.8% between 61 and 70 (3)  0.0% between 71 and 80 (0)  1.2% between 81 and 90 (2)  1.8% between 91 and 100 (3)  1.8% between 101 and 200 (3)  8.8% Did not state (15) |
| 4. Number of specimens sampled per parent colony | 171 | 78.4% Reported (Mean ± 1SD = 8 ± 13; Median = 3) (134)  61.4% between 1 and 10 (105)  8.8% between 11 and 20 (15)  4.1% between 21 and 30 (7)  0.6% between 31 and 40 (1)  1.2% between 41 and 50 (2)  1.2% between 51 and 60 (2)  0.6% between 61 and 70 (1)  0.6% between 71 and 80 (1)  21.6% Did not state (37) |
| 5. Total number of specimens collected per study | 171 | 96.5% Reported (Mean ± 1SD = 133 ± 194; Median = 60) (165)  44.4% between 1 and 50 (76)  15.8% between 51 and 100 (27)  17.5% between 101 and 200 (30)  8.2% between 201 and 300 (14)  5.3% between 301 and 400 (9)  2.3% between 401 and 500 (4)  2.9% more than 500 (5)  3.5% Did not state (6) |
| 6. Total number of specimens collected per year |  | Year  2014 (Jan – Dec) = 2,233  2015 (Jan – Dec) = 4,334  2016 (Jan – Dec) = 4,845  2017 (Jan – Dec) = 2,354  2018 (Jan – Dec) = 2,168  2019 (Jan – Dec) = 4,016  2012 (Jan – Aug) = 1,940  TOTAL (Jan 2014 – August 2020) = 21,890 |
| **3) SPECIMEN COLLECTION** | | |
| 1. Type of specimen collected from reef | 171 | 89.5% Fragments (153)  6.4% Skeletal cores (11)  2.9% Mucus (5)  0.6% Tissue (1)  0.6% Gametes (1) |
| 2. Tool(s) used to collect specimens | 171 | 36.3% Reported (68)  15.2% Hammer and Chisel (26)  7.6% Drill (13)  4.1% Bone cutters (7)  2.3% Syringe (4)  2.3% Pliers (4)  1.8% Mucus tube (3)  1.8% Filter / mesh (3)  1.2% Chisel (2)  1.2% Wire cutters (2)  1.2% Corer / hole punch (2)  0.6% Cotton swab (1)  0.6% Razor blade (1)  60.2% Not reported (103) |
| 3. If sterile techniques were used | 171 | 2.3% Sterile tools (4)  2.9% Sterile storage containers (5)  94.7% Not reported (162) |
| 4. Size of parent colony | 171 | 8.2% Reported some measure of size (14)  2.9% Height (Mean ± 1SD: 71.4 ± 72.0 cm) (5)  5.3% Diameter (Mean ± 1SD: 74.4 ± 57.8 cm) (9)  91.8% Not reported (157) |
| 5. Size of specimen | 171 | 63.2% Reported some measure of size (108)  35.1%^b^ Height (Mean ± 1SD: 4.25 ± 2.10 cm) (60)  22.2%^b^ Diameter (Mean ± 1SD: 5.97 ± 5.88 cm) (38)  14.6%^b^ Surface area (Mean ± 1SD: 6.73 ± 7.62 cm^2^) (25)  4.1% Not applicable (7)  32.7% Did not report any measure of size (56) |
| 6. Specimen transportation methods | 171 | 29.8% Reported (51)  70.2% Not reported (120) |
| 7. Specimen transportation duration | 171 | 5.3% Reported (Mean ± 1SD: 152 ± 216 min) (9)  94.7% Not reported (162) |
| 8. Type of specimen collected post-experiment | 105 | Bleaching experiment studies:  88.6% Fragments (93)  6.7% Tissue (7)  2.9% Mucus (3)  1.9% Symbiodiniaceae cells (2) |
| **4) SPECIMEN SACRIFICE** ^c^ | | |
| 1. Method of specimen sacrifice | 197 | 36.5% Freezing (72)  23.4% Snap frozen using liquid nitrogen (46)  12.7% Frozen using conventional freezer (25)  5.6% Frozen to -80 ℃ (11)  4.1% Frozen to -20 ℃ (8)  0.5% Frozen to -50 ℃ (1)  2.5% Frozen, but temperature not specified (5)  0.5% Cryopreservation (1)  31.0% Chemical (61)  21.2% Preservative (42)  9.6% Ethanol (19)  7.1% RNA buffer (14)  2.0% DNA buffer (4)  1.5% Dimethyl sulfoxide [DMSO] (3)  1.0% Methanol (2)  7.5% Fixative (15)  2% Glutaraldehyde (4)  2% Formalin (4)  1.5% Paraformaldehyde (3)  1.0% Formaldehyde (2)  0.5% Mercuric chloride [Zenker’s sol.] (1)  0.5% Fixed, but chemical not specified (1)  1.5% Bleach (3)  0.5% Hydrochloric acid (1)  21.8% Mechanical (43)  18.3% Airbrushed (36)  3.6% Ground (7)  10.7 % Not reported / was not clear (21) |
| **5) SPECIMEN PROCESSING** ^c^ | | |
| 1. Post-sacrifice processing techniques | 197 | 96.5% Reported (190)  3.5% Not reported (7) |
| 2. Airbrushing methods | 97 | Studies which airbrushed fragments:  44.3% Airbrushed with filtered salt water (35)  17.7% Airbrushed with buffer (14)  5.1% Airbrushed with air only (4)  6.3% Airbrushed with deionized freshwater (5)  8.9% Airbrushed, but medium not specified (7)  10.1% Water-piked with filtered seawater (8)  1.3% Water-piked with buffer (1)  6.3% Water-piked, but medium not specified (5) |
| 3. Homogenization methods | 76 | Studies which homogenized specimens ^d^  20.8% Electric homogenizer (16)  19.5% Mortar and pestle (15)  7.8% Potter Elvehjem homogenizer (6)  6.5% Bead homogenizer (5)  6.5% Disperser (5)  5.2% Handheld glass homogenizer (4)  5.2% Domestic blender (4)  1.3% Tissue lyser (1)  1.3% Razor blade (1)  1.3% Rotor-strator homogenizer (1)  1.3% Syringe (1)  23.4% Homogenized, but method not specified (18) |
| 4. Short-term storage temperature | 197 | 58.9% Reported (116)  25.4% stored at -80 ℃ (50)  0.5% stored at -50 ℃ (1)  1.5% stored at -30 ℃ (3)  13.2% stored at -20 ℃ (26)  8.6% stored at +4 ℃ (17)  1.5% stored at +25 ℃ (3)  8.1% stored at multiple temperatures (16)  41.1% Not reported (81) |
| **6) DOWNSTREAM ANALYSES** | | |
| 1. Type of downstream analyses conducted ^e^ | 197 | A) PHYSIOLOGY  28.4% Chlorophyll concentration (56)  18.8% Total soluble protein (37)  7.6% Total lipid and lipid classes (15)  6.6% Skeletal isotopes and elements (13)  3.6% Tissue isotopes (7)  3.0% Carbohydrates (6)  2.5% Biomass (5)  2.5% Pigment analysis (HPLC) (5)  B) -OMICS  28.9% Symbiodiniaceae identification (57)  12.7% Transcriptomics and gene expression (25)  10.7% Microbiome (21)  7.6% Enzymatic assays (15)  3.0% Host genotyping (6)  2.5% Protein stress biomarkers (5)  2.5% Reactive oxygen/nitrogen species (5)  2.5% DMSP/DMSO concentration (5)  2.0% Metabolomics (4)  2.0% Immunological/ Vibrio assays (4)  1.0% Proteomics (2)  1.0% Metagenomics (2)  C) MICROSCOPY AND IMAGING  38.6% Symbiodiniaceae density and mitotic index (76)  5.1% X-ray imaging (10)  2.5% Histology (5)  2.5% Color and morphology image analysis (5)  2.0% Epifluorescent microscopy (4)  2.0% Transmission electron microscopy (4)  1.5% Computerized tomography (3)  1.0% Nanoscale secondary ion mass spectrometry (2)  1.0% Scanning electron microscopy (2) |
| 2. Number of downstream analyses conducted | 197 | 42.1% one downstream analysis (83)  30.5% two downstream analyses (60)  14.7% three downstream analyses (29)  7.1% four downstream analyses (14)  5.6% five or more downstream analyses (11) |
| ^a^ percentages sum > 100 as some studies collected more than coral species  ^b^ percentages sum > 63% as some studies reported more than one metric of size  ^c^ 22 publications sacrificed and preserved specimens using two or more different methods. In these cases, all methods were recorded, thus increasing the sample size from 171 to 197 for specimen preservation and specimen processing.  ^d^ Only 13.2% (n = 10) specified the duration of homogenization  ^e^ percentages sum > 100 as some studies conducted more than one downstream analysis | | |
