# Supplementary material for: A review of coral bleaching specimen collection, preservation, and laboratory processing methods
Source: PeerJ. 2021 Jul 8;9:e11763. doi: 10.7717/peerj.11763 (PMC8272927; doi:10.7717/peerj.11763)
Supplement: Supplemental Information 6 [file peerj-09-11763-s006.docx]

| Name of Journal | Number of studies | Percent of studies (out of 161) |
| --- | --- | --- |
| Coral Reefs | 19 | 11.8 |
| Scientific Reports | 15 | 9.3 |
| PLOS One | 9 | 5.6 |
| Global Change Biology | 8 | 5.0 |
| Frontiers in Marine Science | 6 | 3.7 |
| Journal of Marine Biology & Ecology | 6 | 3.7 |
| Frontiers in Microbiology | 5 | 3.1 |
| Marine Biology | 5 | 3.1 |
| Marine Pollution Bulletin | 5 | 3.1 |
| ISME Journal | 4 | 2.5 |
| Molecular Ecology | 4 | 2.5 |
| Peer J | 4 | 2.5 |
| Journal of Experimental Biology | 3 | 1.9 |
| Marine Environmental Research | 3 | 1.9 |
| Marine Ecology Progress Series | 3 | 1.9 |
| Biological Bulletin | 2 | 1.2 |
| Ecosphere | 2 | 1.2 |
| Environmental Microbiology | 2 | 1.2 |
| Frontiers in Physiology | 2 | 1.2 |
| Geochemistry, Geophysics, Geosystems | 2 | 1.2 |
| Journal of Marine Science & Engineering | 2 | 1.2 |
| Journal of Phycology | 2 | 1.2 |
| Journal of The Marine Biological Association of The United Kingdom | 2 | 1.2 |
| Marine Biology Research | 2 | 1.2 |
| Marine Ecology | 2 | 1.2 |
| Metabolomics | 2 | 1.2 |
| Nature Communications | 2 | 1.2 |
| Science Advances | 2 | 1.2 |
| Anais Da Academia Brasiliera De Ciencias | 1 | 0.6 |
| Applied Ecology & Environmental Research | 1 | 0.6 |
| Aquatic Toxicology | 1 | 0.6 |
| Biogeosciences | 1 | 0.6 |
| BMC Biology | 1 | 0.6 |
| BMC Genomics | 1 | 0.6 |
| Cell Stress & Chaperones | 1 | 0.6 |
| Comparative Biochemistry & Physiology - Part A | 1 | 0.6 |
| Current Biology | 1 | 0.6 |
| Developmental & Comparative Immunology | 1 | 0.6 |
| Diseases of Aquatic Organisms | 1 | 0.6 |
| Ecological Indicators | 1 | 0.6 |
| Ecology & Evolution | 1 | 0.6 |
| Environmental Science & Pollution Research | 1 | 0.6 |
| FEMS Microbiology Ecology | 1 | 0.6 |
| Fish & Shellfish Immunology | 1 | 0.6 |
| Frontiers in Genetics | 1 | 0.6 |
| Geochemical Journal | 1 | 0.6 |
| Geophysical Research Letters | 1 | 0.6 |
| Ices Journal of Marine Science | 1 | 0.6 |
| Journal of Geophysical Research | 1 | 0.6 |
| Limnology & Oceanography | 1 | 0.6 |
| Microbial Ecology | 1 | 0.6 |
| Microbiology Open | 1 | 0.6 |
| Microbiome | 1 | 0.6 |
| Microorganisms | 1 | 0.6 |
| Molecular & Cellular Proteomics | 1 | 0.6 |
| Molecular Ecology Resources | 1 | 0.6 |
| Proceedings of The Royal Society B | 1 | 0.6 |
| Regional Studies in Marine Science | 1 | 0.6 |
| Remote Sensing | 1 | 0.6 |
| Reproduction, Fertility, & Development | 1 | 0.6 |
| Royal Society Open Science | 1 | 0.6 |
| Science | 1 | 0.6 |
| Systematic & Applied Microbiology | 1 | 0.6 |
| Water | 1 | 0.6 |
