# Supplementary material for: A review of coral bleaching specimen collection, preservation, and laboratory processing methods
Source: PeerJ. 2021 Jul 8;9:e11763. doi: 10.7717/peerj.11763 (PMC8272927; doi:10.7717/peerj.11763)
Supplement: Supplemental Information 7 — Details regarding exactly which studies (i.e., author, year, and study title) used which species can be found in Supplement 2. [file peerj-09-11763-s007.docx]

| Family | Genus | Species | Number of studies | Percent of studies (out of 171) |
| --- | --- | --- | --- | --- |
| Acroporidae | *Acropora* | *aculeus* | 1 | 0.6 |
| Acroporidae | *Acropora* | *arabiensis* | 3 | 1.8 |
| Acroporidae | *Acropora* | *aspera* | 8 | 4.7 |
| Acroporidae | *Acropora* | *brueggemanni* | 1 | 0.6 |
| Acroporidae | *Acropora* | *cervicornis* | 2 | 1.2 |
| Acroporidae | *Acropora* | *digitifera* | 2 | 1.2 |
| Acroporidae | *Acropora* | *downingi* | 6 | 3.5 |
| Acroporidae | *Acropora* | *eurystoma* | 1 | 0.6 |
| Acroporidae | *Acropora* | *globiceps* | 1 | 0.6 |
| Acroporidae | *Acropora* | *hemprichii* | 1 | 0.6 |
| Acroporidae | *Acropora* | *humilis* | 1 | 0.6 |
| Acroporidae | *Acropora* | *hyacinthus* | 4 | 2.3 |
| Acroporidae | *Acropora* | *intermedia* | 3 | 1.8 |
| Acroporidae | *Acropora* | *microphthalma* | 1 | 0.6 |
| Acroporidae | *Acropora* | *millepora* | 20 | 11.7 |
| Acroporidae | *Acropora* | *muricata* | 7 | 4.1 |
| Acroporidae | *Acropora* | *palmata* | 1 | 0.6 |
| Acroporidae | *Acropora* | *pruinosa* | 1 | 0.6 |
| Acroporidae | *Acropora* | *samoensis* | 1 | 0.6 |
| Acroporidae | *Acropora* | *selago* | 2 | 1.2 |
| Acroporidae | *Acropora* | *solitaryensis* | 1 | 0.6 |
| Acroporidae | *Acropora* | *sp.* | 2 | 1.2 |
| Acroporidae | *Acropora* | *tenuis* | 3 | 1.8 |
| Acroporidae | *Acropora* | *valenciennesi* | 1 | 0.6 |
| Acroporidae | *Isopora* | *palifera* | 2 | 1.2 |
| Acroporidae | *Montipora* | *aequituberculata* | 2 | 1.2 |
| Acroporidae | *Montipora* | *capitata* | 5 | 2.9 |
| Acroporidae | *Montipora* | *digitata* | 9 | 5.3 |
| Acroporidae | *Montipora* | *sp.* | 2 | 1.2 |
| Acroporidae | *Montipora* | *truncata* | 1 | 0.6 |
| Agariciidae | *Agaricia* | *lamarki* | 1 | 0.6 |
| Agariciidae | *Agaricia* | *sp.* | 1 | 0.6 |
| Agariciidae | *Gardineroseris* | *planulata* | 1 | 0.6 |
| Agariciidae | *Pachyseris* | *rugosa* | 2 | 1.2 |
| Agariciidae | *Pavona* | *decussata* | 4 | 2.3 |
| Caryophylliidae | *Caryophyllia* | *inornata* | 1 | 0.6 |
| Coscinaraeidae | *Anomastrea* | *sp.* | 1 | 0.6 |
| Coscinaraeidae | *Coscinaraea* | *columna* | 2 | 1.2 |
| Coscinaraeidae | *Coscinaraea* | *mcneilli* | 1 | 0.6 |
| Coscinaraeidae | *Coscinaraea* | *sp.* | 1 | 0.6 |
| Dendrophylliidae | *Astroides* | *calycularis* | 1 | 0.6 |
| Dendrophylliidae | *Balanophyllia* | *europaea* | 3 | 1.8 |
| Dendrophylliidae | *Duncanopsammia* | *axifuga* | 1 | 0.6 |
| Dendrophylliidae | *Leptopsammia* | *pruvoti* | 1 | 0.6 |
| Dendrophylliidae | *Turbinaria* | *peltata* | 3 | 1.8 |
| Dendrophylliidae | *Turbinaria* | *reniformis* | 3 | 1.8 |
| Dendrophylliidae | *Turbinaria* | *sp.* | 2 | 1.2 |
| Euphyllidae | *Galaxea* | *acrhelia* | 1 | 0.6 |
| Euphyllidae | *Galaxea* | *astreata* | 1 | 0.6 |
| Euphyllidae | *Galaxea* | *fasicularis* | 7 | 4.1 |
| Faviidae | *Cladocora* | *caespitosa* | 3 | 1.8 |
| Faviidae | *Leptastrea* | *bottae* | 1 | 0.6 |
| Faviidae | *Leptastrea* | *sp.* | 1 | 0.6 |
| Faviidae | *Leptastrea* | *transversa* | 1 | 0.6 |
| Fungia | *Fungia* | *fungites* | 1 | 0.6 |
| Fungia | *Fungia* | *repanda* | 1 | 0.6 |
| Fungia | *Fungia* | *scutaria* | 2 | 1.2 |
| Meandrinidae | *Meandrina* | *danae* | 1 | 0.6 |
| Meandrinidae | *Meandrina* | *jacksoni* | 1 | 0.6 |
| Meandrinidae | *Meandrina* | *meandrites* | 1 | 0.6 |
| Merulinidae | *Coelastrea* | *aspera* | 2 | 1.2 |
| Merulinidae | *Cyphastrea* | *chalcidicum* | 1 | 0.6 |
| Merulinidae | *Cyphastrea* | *microphthalma* | 5 | 2.9 |
| Merulinidae | *Cyphastrea* | *serailia* | 1 | 0.6 |
| Merulinidae | *Cyphastrea* | *sp.* | 1 | 0.6 |
| Merulinidae | *Dipsastraea* | *favus* | 3 | 1.8 |
| Merulinidae | *Dipsastraea* | *pallida* | 3 | 1.8 |
| Merulinidae | *Dipsastraea* | *sp.* | 2 | 1.2 |
| Merulinidae | *Echinopora* | *gemmacea* | 1 | 0.6 |
| Merulinidae | *Echinopora* | *mammiformis* | 1 | 0.6 |
| Merulinidae | *Favites* | *abdita* | 1 | 0.6 |
| Merulinidae | *Favites* | *colemani* | 1 | 0.6 |
| Merulinidae | *Favites* | *pentagona* | 3 | 1.8 |
| Merulinidae | *Goniastrea* | *edwardsi* | 1 | 0.6 |
| Merulinidae | *Goniastrea* | *minuta* | 1 | 0.6 |
| Merulinidae | *Leptoria* | *phrygia* | 2 | 1.2 |
| Merulinidae | *Orbicella* | *annularis* | 2 | 1.2 |
| Merulinidae | *Orbicella* | *arbuscula* | 1 | 0.6 |
| Merulinidae | *Orbicella* | *faveolata* | 11 | 6.4 |
| Merulinidae | *Platygyra* | *carnosus* | 1 | 0.6 |
| Merulinidae | *Platygyra* | *crosslandi* | 1 | 0.6 |
| Merulinidae | *Platygyra* | *daedalea* | 7 | 4.1 |
| Merulinidae | *Platygyra* | *sp.* | 1 | 0.6 |
| Merulinidae | *Platygyra* | *verucosa* | 1 | 0.6 |
| Montastraeidae | *Montastrea* | *cavernosa* | 5 | 2.9 |
| Mussidae | *Diploria* | *labyrinthiformis* | 2 | 1.2 |
| Mussidae | *Favia* | *pallida* | 1 | 0.6 |
| Mussidae | *Mussismilia* | *harttii* | 2 | 1.2 |
| Mussidae | *Mussismilia* | *hispida* | 1 | 0.6 |
| Mussidae | *Pseudodiploria* | *strigosa* | 6 | 3.5 |
| Oculinidae | *Oculina* | *patagonica* | 3 | 1.8 |
| Plesiastreidae | *Plesiastrea* | *curta* | 1 | 0.6 |
| Plesiastreidae | *Plesiastrea* | *versipora* | 3 | 1.8 |
| Pocilloporidae | *Pocillopora* | *acuta* | 1 | 0.6 |
| Pocilloporidae | *Pocillopora* | *damicornis* | 18 | 10.5 |
| Pocilloporidae | *Pocillopora* | *eydouxi* | 1 | 0.6 |
| Pocilloporidae | *Pocillopora* | *meandrina* | 2 | 1.2 |
| Pocilloporidae | *Pocillopora* | *sp.* | 1 | 0.6 |
| Pocilloporidae | *Pocillopora* | *verucosa* | 5 | 2.9 |
| Pocilloporidae | *Seriatopora* | *caliendrum* | 3 | 1.8 |
| Pocilloporidae | *Seriatopora* | *hystrix* | 5 | 2.9 |
| Pocilloporidae | *Stylophora* | *pistillata* | 18 | 10.5 |
| Pocilloporidae | *Stylophora* | *sp.* | 2 | 1.2 |
| Poritidae | *Goniopora* | *columna* | 1 | 0.6 |
| Poritidae | *Goniopora* | *lobata* | 4 | 2.3 |
| Poritidae | *Porites* | *astreoides* | 8 | 4.7 |
| Poritidae | *Porites* | *compressa* | 2 | 1.2 |
| Poritidae | *Porites* | *cylindrica* | 6 | 3.5 |
| Poritidae | *Porites* | *divaricata* | 3 | 1.8 |
| Poritidae | *Porites* | *evermanni* | 1 | 0.6 |
| Poritidae | *Porites* | *harrisoni* | 3 | 1.8 |
| Poritidae | *Porites* | *heronensis* | 1 | 0.6 |
| Poritidae | *Porites* | *lobata* | 6 | 3.5 |
| Poritidae | *Porites* | *lutea* | 9 | 5.3 |
| Poritidae | *Porites* | *porites* | 1 | 0.6 |
| Poritidae | *Porites* | *rus* | 1 | 0.6 |
| Poritidae | *Porites* | *sp.* | 13 | 7.6 |
| Poritidae | *Porties* | *nodifera* | 2 | 1.2 |
| Psammocoridae | *Psammocora* | *contigua* | 2 | 1.2 |
| Siderastreidae | *Siderastrea* | *radians* | 1 | 0.6 |
| Siderastreidae | *Siderastrea* | *siderea* | 5 | 2.9 |
| Siderastreidae | *Siderastrea* | *sp.* | 1 | 0.6 |
